# Supplementary material for: FerriTag is a new genetically-encoded inducible tag for correlative light-electron microscopy
Source: Nat Commun. 2018 Jul 4;9:2604. doi: 10.1038/s41467-018-04993-0 (PMC6031641; doi:10.1038/s41467-018-04993-0)
Supplement: Supplementary file 1 — Supplementary Information [file 41467_2018_4993_MOESM1_ESM.pdf]

Supplementary Information for FerriTag is a new genetically-encoded inducible tag for correlative light-electron microscopy

Nicholas I. Clarke and Stephen J. Royle*

Centre for Mechanochemical Cell Biology, Warwick Medical School, Gibbet Hill Road, Coventry, CV4 7AL, UK

*Corresponding author: s.j.royle@warwick.ac.uk

Supplementary Note 1: Optimization of FerriTag strategy

As described in the main paper, direct fusion of a ferritin tag to a protein-of-interest is disruptive. Direct fusion of ferritin heavy chain (FTH1) to a mitochondrially targeted protein fragment (Tom70p) resulted in aggregation of mitochondria in HeLa cells (Supplementary Figure 1A). Expressing FRB-mCherry-FTH1 alone was also unsuccessful for inducible FerriTagging due to aggregation of ferritin particles before and after addition of rapamycin (Supplementary Figure 1B). The key to successful FerriTagging was the dilution of FRB-mCherry-tagged FTH1 subunits with untagged FTL subunits (Supplementary Figure 1C). A 1:4 dilution of DNA for transfection resulted in a homogeneous distribution of FRB-mCherry-FTH1 prior to addition of rapamycin and in the effective labeling of the protein-of-interest after application.

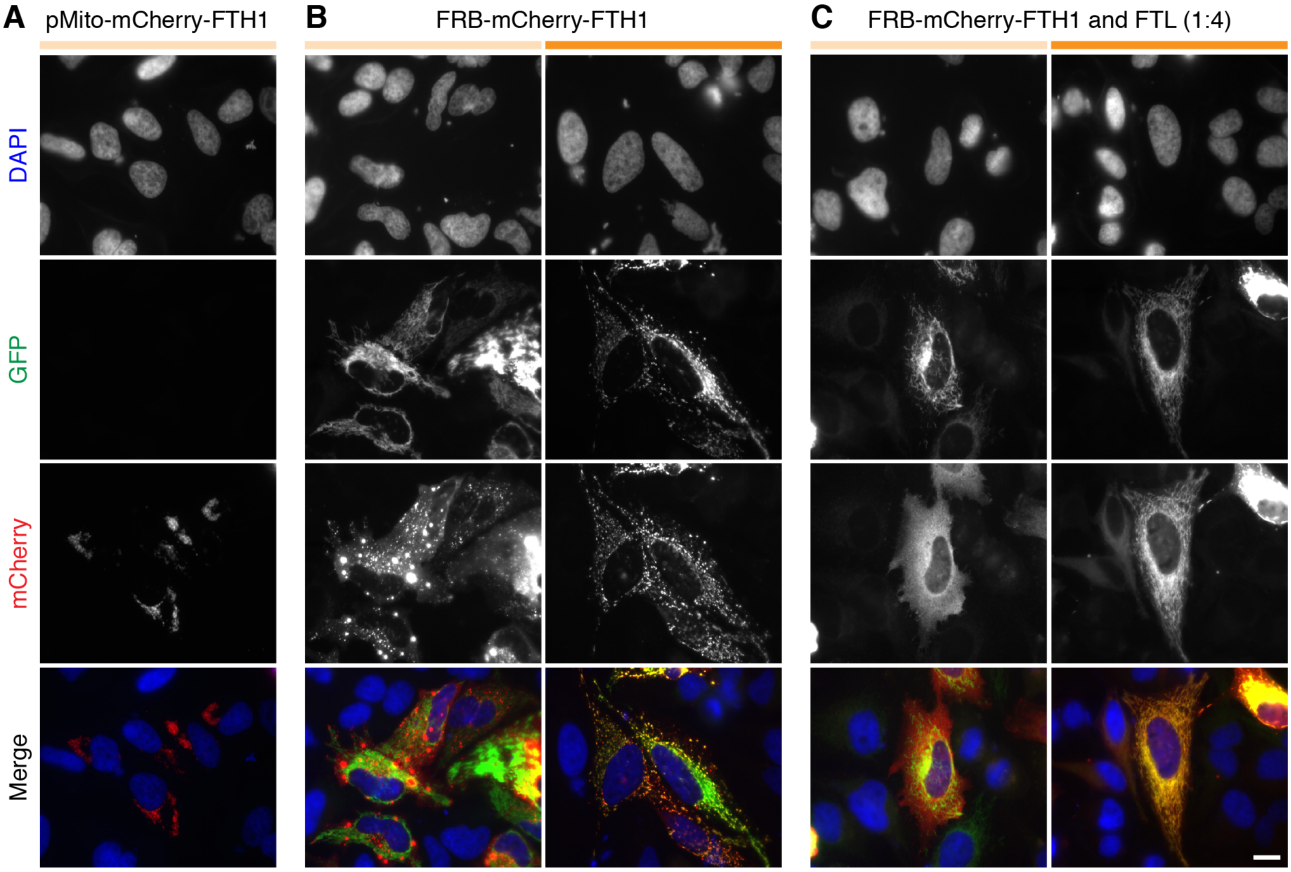

Supplementary Figure 1: Optimizing FerriTag expression.
(**A**) Direct tagging of a mitochondrial protein with ferritin (FTH1) causes aggregation of mitochondria. HeLa cells expressing mCherry-FTH1 fused to the mitochondrial targeting sequence of Tom70p.
(**B**) Expressing FRB-mCherry-FTH1 only with FKBP-GFP-MAO, results in aggregates of FRB-mCherry-FTH1 before rapamycin. After rapamycin treatment the mitochondria become mislocalized and aggregated.
(**C**) Expressing FerriTag (FRB-mCherry-FTH1 and FTL, 1:4) results in successful labeling of mitochondria. No aggregation is seen before or after the addition of rapamycin. Rapamycin addition is indicated by filled orange bar. Scale bar 10 µm.

Supplementary Note 2: Does the FerriTagging protocol disrupt normal cell biology?

Loading FerriTag with iron is non-toxic to cells

In order to increase the electron density of FerriTag, an iron-loading step is required. Previous work indicated that 72 h incubation with 3.3 mM $FeSO_4$ had adverse effects on HeLa cells[1]. It is not clear what effect, if any, shorter incubations or lower concentrations of iron have on HeLa cells. We determined the toxicity of iron-rich media for a range of supplementation concentrations over periods of up to 48 h (Supplementary Figure 2). We observed minimal toxicity of HeLa cells cultured for 16 h in media supplemented with $FeSO_4$ to a final concentration of 1 mM. Accordingly, this concentration was used for our FerriTagging protocol. Throughout this study we didn't find evidence for perturbation of ultrastructure as a result of the iron-loading procedure.

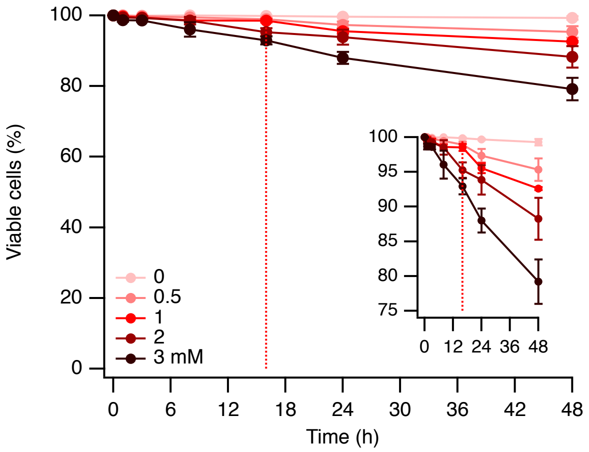

Supplementary Figure 2: Quantification of cell viability in iron-supplemented media.
HeLa cells were incubated at 37 °C in full culture media, supplemented with the indicated concentrations of $FeSO_4$ for durations up to 48 h. Cell viability was assessed by trypan blue exclusion. Markers indicate mean ± SD from three experiments. Minimal toxicity was observed at 16 h for all concentrations. Dashed red line indicates the 1 mM supplementation condition used in the paper.

Localization of cellular markers is unaffected by the FerriTagging procedure

It is possible that either the iron-loading procedure or the addition of rapamycin itself, used in the FerriTagging procedure may disrupt cellular structures. To investigate this possibility, we transfected HeLa cells with 12 different XFP-tagged cDNAs and simply looked at their subcellular distribution in cells with or without iron-loading (1 mM, 16 h) and rapamycin treatment (200 nM, 5 min). The distribution of actin cytoskeleton (mNeonGreen-Actin-C-18 or mNeonGreen-alpha-Actinin-19), lysosomes (mNeonGreen-LAMP1-20), mitochondria (FKBP-GFP-MAO), caveolae (mNeonGreen-Caveolin-C-10), endoplasmic reticulum (pAc-GFP-Sec61beta), clathrin coated structures (mNeonGreen-Clathrin-15), microtubules (GFP-EB1, mNeonGreen-Tubulin-C-35), intermediate filaments (mNeonGreen-Vimentin-7), nuclei (mNeonGreen-H2B-C-10) and adhesion complexes (mNeonGreen-Zyxin-6) was examined (Supplementary Figure 3). No evidence for disruption of any of these structures was observed in HeLa cells following the iron-loading and rapamycin treatment conditions used for FerriTagging.

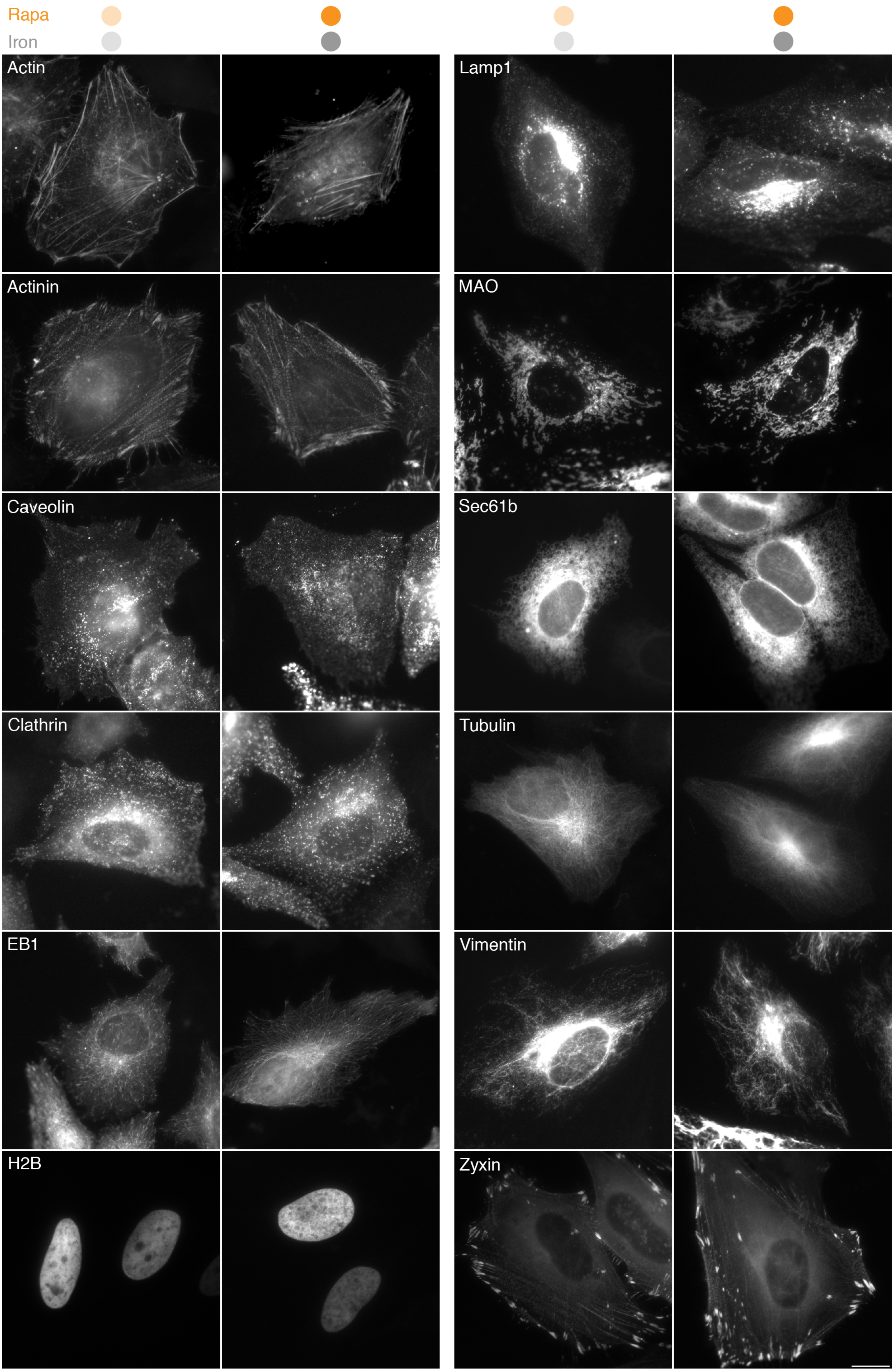

Supplementary Figure 3: Localization of twelve cell markers is unaltered after iron-loading and rapamycin treatment.
Micrographs of HeLa cells transiently transfected with the indicated XFP-tagged proteins. Cells were loaded with iron (1 mM, 16 h) and treated with rapamycin (200 nM, 5 min) before fixation. Scale bar, 10 μm.

FerriTagging without induction of autophagy

Prolonged application of rapamycin induces autophagy[2]. This is a potential concern for FerriTagging, or indeed any experiment that uses rapamycin to heterodimerize FKBP and FRB domains[3,4]. To test the time course of autophagy induction by application of 200 nM rapamycin in HeLa cells, we imaged the generation of mNeonGreen-LC3B puncta (Supplementary Figure 4A). While puncta could be observed after extended rapamycin applications (>4 h), no puncta were observed after 10 min application. Typical FerriTagging experiments are concluded by fixation after 5 min and so it is unlikely that autophagy induction is an issue. In experiments where FerriTagged proteins need to be tracked in live cells over much longer periods, then an alternative to rapamycin should be used. Accordingly, we generated a mutant version of FerriTag where a T2098L mutation was introduced into FRB-mCherry-FTH1 and expressed at a 4:1 ratio with unlabeled FTL. The T2098L mutation allows rapalogs to bind FRB – whereas these drugs cannot bind and inhibit mTOR to induce autophagy[5]. FerriTagging of GFP-FKBP-LCa was possible using a Rapalog (AP21967, 1 µM) and the results were similar to the FerriTagging protocol using rapamycin (Supplementary Figure 4B). These experiments suggest that Rapalog is a viable alternative to rapamycin for FerriTagging if an experiment requires prolonged live cell imaging, or in circumstances when autophagic processes may disrupt the experiment.

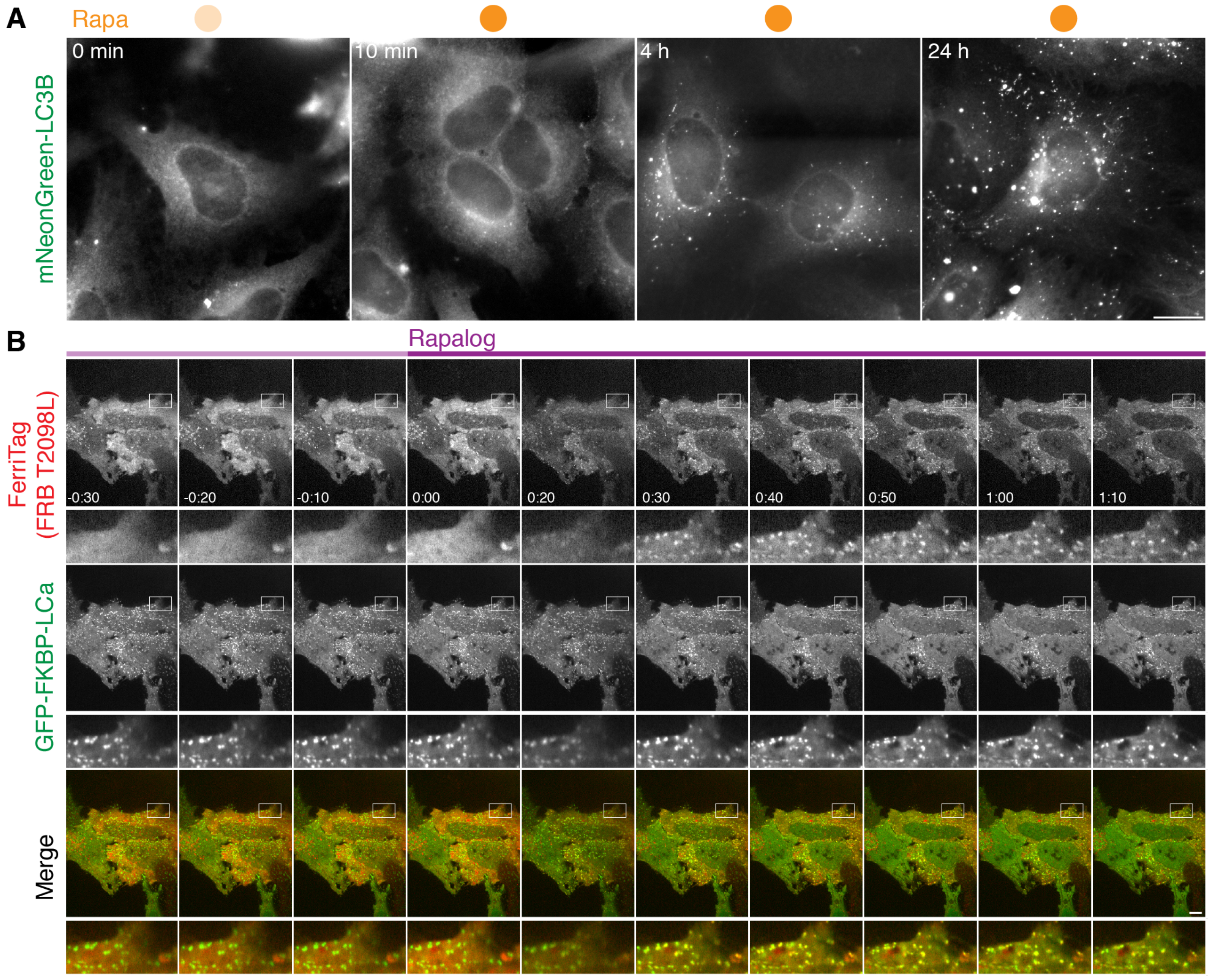

Supplementary Figure 4: FerriTagging without induction of autophagy.
(**A**) Representative micrographs of HeLa cells expressing mNeonGreen-LC3B, treated with rapamycin (200 nM) for the indicated times. FerriTagging is typically done on short time scales before rapamycin induction. Scale bar, 10 µm.
(**B**) FerriTagging of clathrin (GFP-FKBP-LCa) using Rapalog (AP21967, 1 µM) and a T2098L mutant of FerriTag. Scale bar, 10 µm. Zoomed images show 4x expansion.

Supplementary Note 3: Computer Simulations of FerriTagging

In electron micrographs, FerriTag appears as a discrete electron-dense particle corresponding to the ferritin component of the tag. Due to the protein domains used for induction and visualization, this density may be some distance away from the target protein. Determining the average distance of the particle from the target will tell us the "labeling resolution". In the main paper, the outcome of experiments to determine the labeling resolution is reported along with a summary of computer simulations. These simulations are described in detail here. Our best estimate for the labeling resolution of FerriTagging is 10 ± 5 nm.

Experimentally, we could FerriTag CD8-GFP-FKBP, which can be considered as a single point on the plasma membrane. The perpendicular distance from the membrane to the ferritin particle can be measured and this could be used to determine the labeling resolution of FerriTagging. However, this distance was unlikely to correspond directly to the labeling resolution because of four variables.

1. Length state of FerriTag
2. Pose of FerriTag
3. Sampling via sectioning
4. Measurement and associated error from the EM images

Modeling FerriTag as a ball-and-chain We wrote a computational model to simulate the experimental data to understand the relationship between the experimental observation and the real proximity of FerriTag to the target protein. To do this, we set the point of FerriTagging to be the origin on an xy plane at $z = 0$, and modeled FerriTag as a ball-and-chain, building in length state, pose, sampling and error in the simulation.

Length state of FerriTag What is the maximum distance that a ferritin particle may be from the target protein? To answer this, PDB files corresponding to structures for EGFP (1EMA), FKBP-rapamycin-FRB (3FAP) and mCherry (2H5Q), and ferritin (2FFX) were assembled co-linearly using PyMol (Supplementary Figure 5). The lengths of the long axis of each were 4.9 nm, 5.3 nm and 4.6 nm for GFP, FKBP-Rapa-FRB, and mCherry, respectively. To allow for linkers, this is a maximum combined length of 15.5 nm. Ferritin is a spherical shell of 24 ferritin light or heavy chain proteins, and has a radius of 6.5 nm. Therefore, the furthest point that the center of the Ferritin particle can be from the end of the protein being FerriTagged is 22 nm.

Due to the flexibility in the linkers between the domains from the target protein to the particle, it is likely that Ferritin is situated at distances shorter than 22 nm. For modeling, the center of the particle should be considered to exist anywhere from 6.5 nm to 22 nm away. We refer to this variable distance as the length state of FerriTag.

Pose of FerriTag It is assumed that FerriTag can be posed in a number of conformations such that the centre of Ferritin corresponds to the spherical coordinate triplet, (r, θ, ϕ), giving the coordinates, $x = r \sin\theta \cos\phi$, $y = r \sin\theta \sin\phi$, and $z = r \cos\theta$, where $r \in (6.5, 22]$, $\theta \in [0, \frac{\pi}{2} - \alpha]$, and $\phi \in [0, 2\pi]$.

Exclusion of α radians from the upper limit of θ is necessary because the outer edge of Ferritin may only touch the xy plane at $z = 0$, but not pass through it. Accordingly, α is calculated by Supplementary Equation 1.

$$\sin\alpha = \frac{6.5}{r} \tag{1}$$

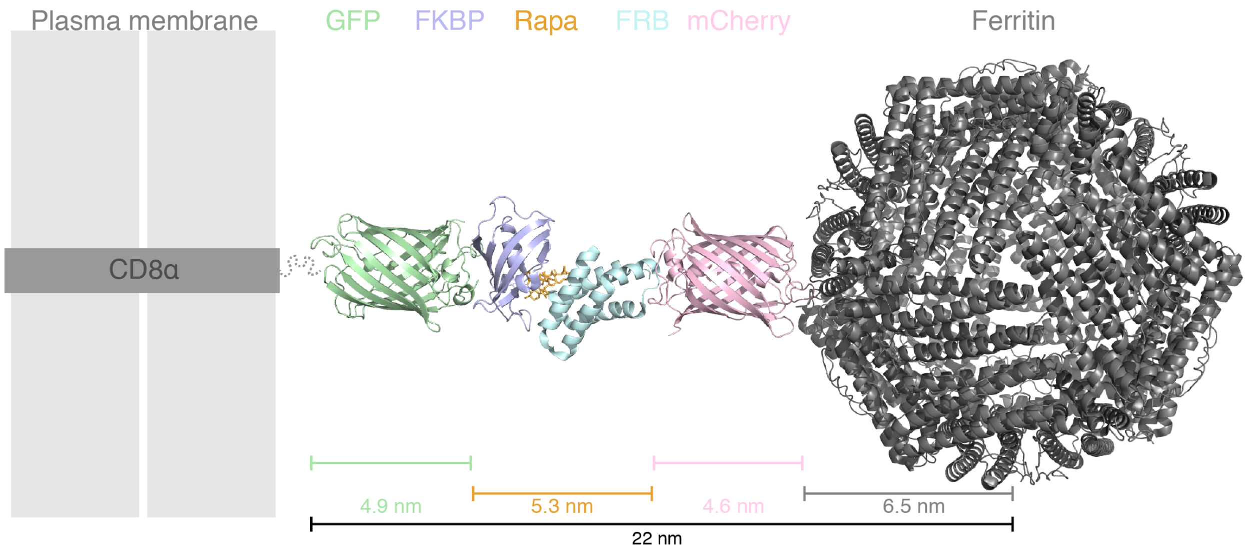

Supplementary Figure 5: Maximum distance of ferritin particle during FerriTagging. FerriTag (FRB-mCherry-FTH1 and FTL) is shown in complex with FKBP bound to rapamycin. The FKBP domain is attached, along with GFP, to the alpha chain of CD8. CD8α is a transmembrane protein which is shown approximately to scale, in the plasma membrane. Protein domains are organized co-linearly with their long axis, giving a total maximum length of 22 nm to the center of the Ferritin particle.

Sampling via sectioning Computer simulations posed the FerriTag in a variety length states and sectioning was simulated by excluding points which fell outside two xz planes, 70 nm apart (corresponding to section thickness) placed randomly on the Y axis in the interval $[70 + \frac{r}{2}, -70 - \frac{r}{2})$. The measurement corresponding to the distance from the plasma membrane to the center of the Ferritin particle was given by the z coordinate.

Measurement error Finally, to simulate experimental and measurement error, a random value from a Gaussian distribution ($\mu = 0$ and $\sigma = 1.5$) was added to the measurement. This is reasonable given the width of the 2D Gaussian distribution observed in Supplementary Figure 8D and because there is a small error associated with locating the plasma membrane in these images.

One iteration of the simulation is shown (Supplementary Figure 6).

Experimental data – FerriTagging CD8-GFP-FKBP

As described in the main paper, the location of ferritin particles was measured as the distance from the center of the particle to the plasma membrane in electron micrographs. The median distance measured experimentally was 9.5 nm. These values were best matched by a random distribution of FerriTag length states ranging from 7 nm to 18 nm (Supplementary Figure 7A). The median simulated value was in reasonable agreement with the experimental data, 10.5 nm. We conclude from these simulations that FerriTag is quite flexible with a maximum length of 18 nm. Note that this maximum does not correspond to the maximum theoretical length state, suggesting that FerriTagged molecules do not exist in the fully extended colinear arrangement depicted above (Supplementary Figure 5). The simulation further indicates that the observed distance in EM images underestimates the true length state by approximately 11 % to 13 % (Supplementary Figure 7B). To demonstrate that FerriTag is not a 22 nm rigid colinear assembly of domains, a simulation with a 22 nm-only ball-and-chain was carried out. This simulation shows a completely different distribution of observed measurements (Supplementary Figure 7C).

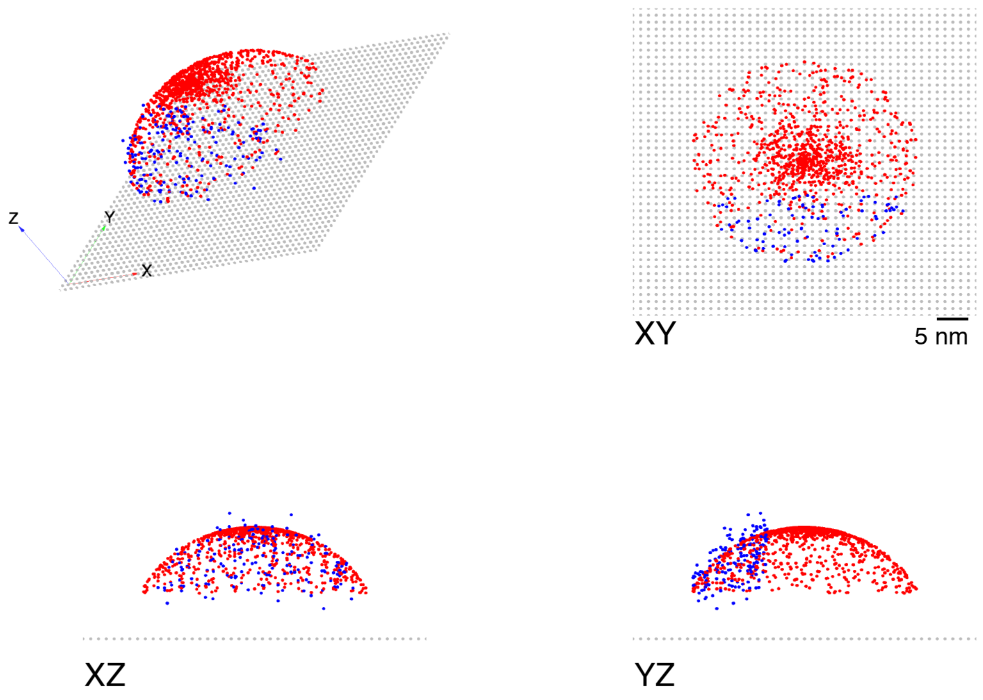

Supplementary Figure 6: Simulation of FerriTagging a single point on a plane. In this simulation, FerriTag has a fixed length state of 16 nm for clarity, 1000 poses are calculated (red). These positions are subdivided as described (blue) to simulate sectioning, leaving 178 spots in this case. Noise is then added to the Z coordinate of these points to simulate error in placing the particle center.

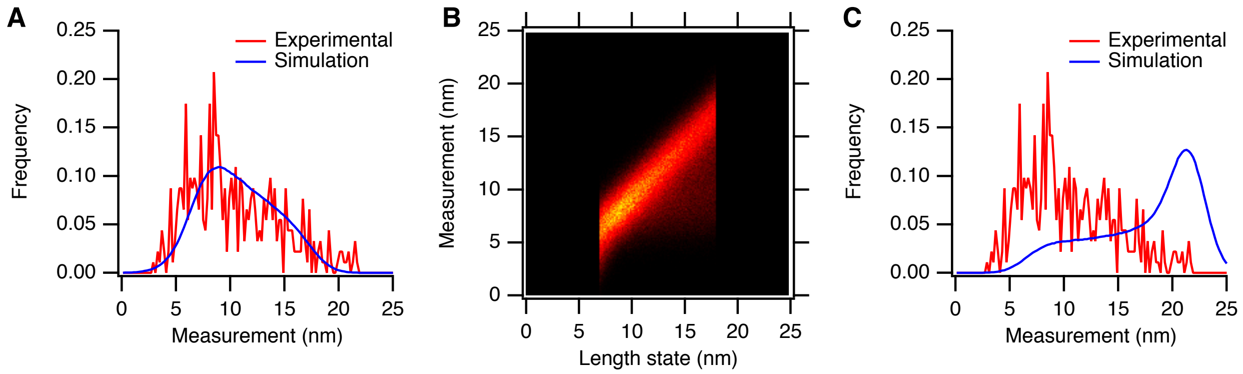

Supplementary Figure 7: Experimental determination and modeling of the labeling resolution of FerriTagging.
(**A**) Histogram of experimental observations (red) with all simulated values overlaid (blue). Simulation using random length states of FerriTag in the range 7 nm to 18 nm. All possible integer bounds from 7 nm to 18 nm were tested (119 simulations). The range of 7 nm to 18 nm had the lowest total sum of squares (TSS) of all simulations.
(**B**) Heat map of the measured distance of FerriTag particle from the plasma membrane as a function of FerriTag length state in the simulation shown. The median simulated observation underestimates the true length by 11% (mean, 13%).
(**C**) Comparison of the observed values with simulation using a 22 nm-only ball-and-chain.

Supplementary Note 4: Signal-to-noise ratio of FerriTagging

To determine the signal-to-noise ratio (SNR) of FerriTagging, we developed a simple image analysis pipeline for the dataset which was used to measure labeling resolution (see below). Since the location of FerriTag particles was determined for this analysis, we used these coordinates to:

1. fit a 2D Gaussian function to each particle
2. determine the SNR

Our aim was to directly determine the SNR but also provide parameters for future automated analysis of FerriTag particles. To do this, the XY coordinates of the Ferritin particle were used to excise a 21×21 pixel box centered on the particle (Supplementary Figure 8A). In turn, this image was fitted with a 2D Gaussian function (Supplementary Equation 2),

$$f(x,y) = z_0 + Aexp\left[\frac{-1}{2(1-cor^2)}\left(\left(\frac{x-x_0}{\sigma_x}\right)^2 + \left(\frac{y-y_0}{\sigma_y}\right)^2 - \frac{2cor(x-x_0)(y-y_0)}{\sigma_x.\sigma_y}\right)\right] \quad (2)$$

where the cross-correlation term, $cor \in [-1, 1]$ (Supplementary Figure 8B).

From 441 particles, accurate fits were retrieved for 389 particles and the coefficients are summarized in Supplementary Figure 8C. SNR is defined by Supplementary Equation 3,

$$SNR = \frac{\mu_{signal}}{\sigma_{background}} \quad (3)$$

Good SNR is difficult to achieve since the cellular ultrastructure needs to be observed in order to do contextual nanoscale mapping of FerriTagged proteins. This necessarily means that contrast is needed in the background of the image, which therefore reduces the SNR. We determined the SNR directly using as the signal a 13×13 nm ROI centered on the x_0 and y_0 values from each fit (Supplementary Figure 8A). The background ROI was a large section of the micrograph avoiding extracellular areas or grid bars. These analyses revealed that FerriTagging, using our current protocol and with manual selection of particles has an SNR of approximately 9:1.

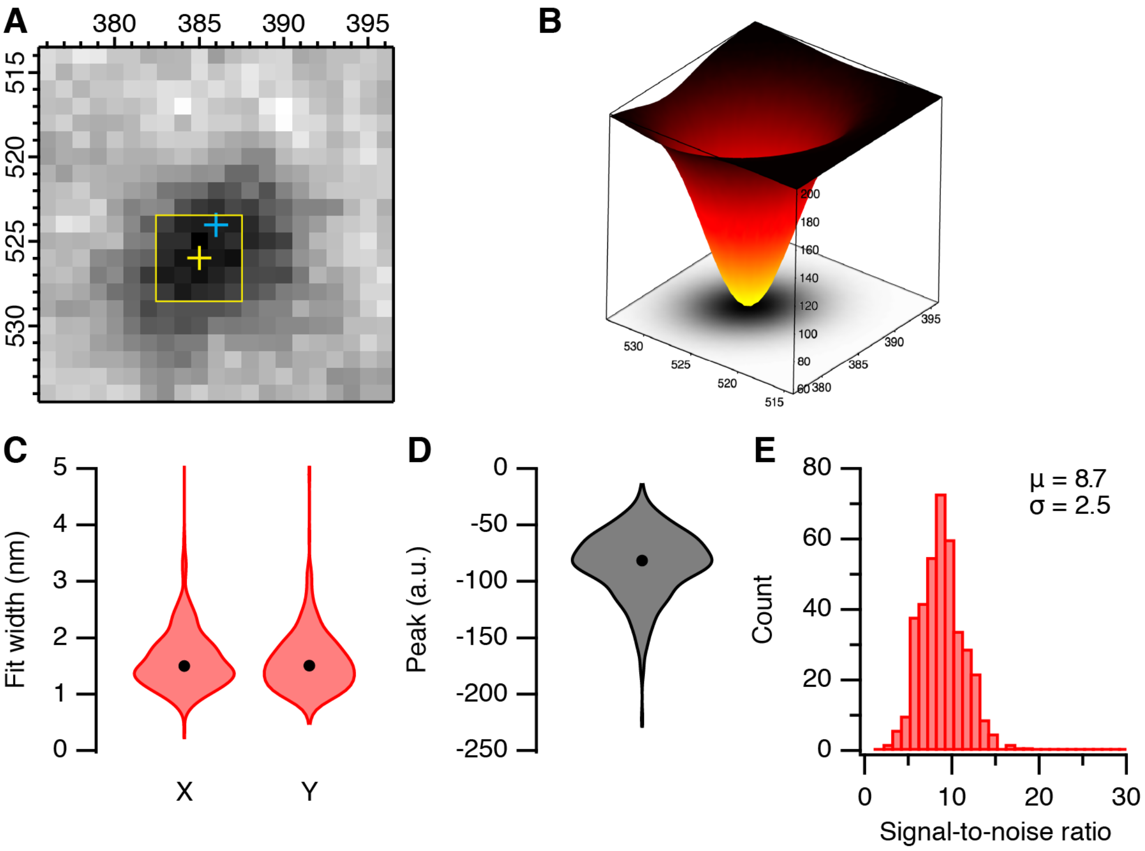

Supplementary Figure 8: Automated fitting in FerriTag images and determination of SNR.
(**A**) Excerpt from an EM micrograph of a Ferritin particle in a cell where CD8-GFP-FKBP was FerriTagged. The location of the centre of the particle was detected by a user (blue cross). The x_0 and y_0 values from each fit (yellow cross) define the centre of an ROI used for measuring the signal of the particle (yellow box).
(**B**) 3D representation of the 2D Gaussian function fitted to the particle shown in A, note the offset of the peak relative to the center of the XY plane.
(**C**) Violin plots of the results of fitting 389 particles showed that the width in X and Y was approximately 1.6 nm.
(**D**) Violin plot of the density of the peak of the fit, which was on average -85 arbitrary units. Dots indicate the sample mean.
(**E**) Histogram of signal-to-noise ratio measured from 389 ferritin particles.

Supplementary Methods

All code used in the manuscript is available in a dedicated repo (`https://github.com/quantixed/FerriTag`). Versions of code used in the paper:

1. `IMODModelAnalysis.ipf` : Analysis of segmented outputs from IMOD for nanoscale mapping

   hash `f26e1a6`.

2. `FerriTag.ipf` : Computer simulations of FerriTagging

   hash `9876d25`.

3. `FTAnalysisSNR.ipf` : SNR Analysis workflow for 2D Gaussian fitting and SNR calculation

   hash `3f3b72f`.

4. `ParseFTData.ipf` : Parsing manually picked FerriTag particles and stereology

   hash `108fdda`.

Supplementary References

[1] Jauregui, H. O., Bradford, W. D., Arstila, A. U., Kinney, T. D. & Trump, B. F. Iron metabolism and cell membranes. iii. iron-induced alterations in hela cells. *Am J Pathol* **80**, 33–52 (1975).

[2] Klionsky, D. J. *et al.* Guidelines for the use and interpretation of assays for monitoring autophagy. *Autophagy* **8**, 445–544 (2012).

[3] Wood, L. A., Larocque, G., Clarke, N. I., Sarkar, S. & Royle, S. J. New tools for "hot-wiring" clathrin-mediated endocytosis with temporal and spatial precision. *J Cell Biol* **216**, 4351–4365 (2017).

[4] Robinson, M. S., Sahlender, D. A. & Foster, S. D. Rapid inactivation of proteins by rapamycin-induced rerouting to mitochondria. *Dev Cell* **18**, 324–31 (2010).

[5] Bayle, J. H. *et al.* Rapamycin analogs with differential binding specificity permit orthogonal control of protein activity. *Chem Biol* **13**, 99–107 (2006).
