# Supplementary material for: FerriTag is a new genetically-encoded inducible tag for correlative light-electron microscopy
Source: Nat Commun. 2018 Jul 4;9:2604. doi: 10.1038/s41467-018-04993-0 (PMC6031641; doi:10.1038/s41467-018-04993-0)
Supplement: Supplementary file 3 — Description of Additional Supplementary Files [file 41467_2018_4993_MOESM3_ESM.pdf]

Description of Additional Supplementary Files

File Name: Supplementary Movie 1
Description: **FerriTagging clathrin in HeLa cells**
Example confocal live cell imaging of rapamycin (200 nM) application to HeLa cells expressing GFP-FKBP-LCa (green, left), FerriTag (red, middle), merge is shown to the right. See Figure 1. Imaging rate, 2 s per frame. Video speed, 10 fps. Rapamycin is applied after 30 s.
